# Supplementary material for: Association of genetic liability to smoking initiation with e-cigarette use in young adults: A cohort study
Source: PLoS Med. 2021 Mar 18;18(3):e1003555. doi: 10.1371/journal.pmed.1003555 (PMC7971530; doi:10.1371/journal.pmed.1003555)
Supplement: S1 Text — (DOCX) [file pmed.1003555.s002.docx]

**Negative control outcomes**

A high number of sexual partners at 23 years was determined using the upper quartile for number of lifetime sexual partners in the ALSPAC sample (11 or more sexual partners). Having been in trouble with the law recently (since 23rd birthday) was self-reported at 24 years. Ever gambling by 24 years was determined using 17 questions about various types of gambling (all questions provided in S1 Table). If the participant had ever engaged in any of these activities, they were considered to have gambled. Enjoying taking risks at 24 years was determined in response to a question asking how much participants agreed with the statement “I quite enjoy taking risks”. Hyperactivity and conduct disorder were measured using the Strengths and Difficulties Questionnaire (SDQ) at 7 years and Oppositional Defiant Disorder (ODD) was measured using the Development and Well-Being Assessment (DAWBA) at 7 years. Scores of 0-5 for hyperactivity are considered ‘normal’, and above 5 is considered borderline/abnormal (i.e., hyperactive/Attention Deficit Hyperactivity Disorder [ADHD]). Scores of 0-2 for conduct disorder determined by teacher/parent are considered normal, and above are considered borderline/abnormal (i.e., conduct disorder). Any awkward symptoms before the age of 7 were considered abnormal (i.e., ODD). SEP was assessed using the 1991 OPCS occupation classification (manual vs non-manual in the parents during pregnancy (32 weeks gestation).
