# Supplementary material for: Association of genetic liability to smoking initiation with e-cigarette use in young adults: A cohort study
Source: PLoS Med. 2021 Mar 18;18(3):e1003555. doi: 10.1371/journal.pmed.1003555 (PMC7971530; doi:10.1371/journal.pmed.1003555)
Supplement: S1 Table — (DOCX) [file pmed.1003555.s003.docx]

| Variable | Question | Possible responses |
| --- | --- | --- |
|  |  |  |
| Ever e-cigarette use by 24 years | Have you ever used/vaped an electronic cigarette (e-cigarette) or other vaping device? | Yes  No |
| Ever smoked by 24 years | Have you ever smoked a whole cigarette (including roll-ups)? | Yes  No |
| Smoking initiation by 24 years | How many cigarettes have you smoked altogether in your lifetime? | Less than 5  5-19  20-49  50-99  100 plus  (Recoded into 2 categories: Initiated smoking [100 plus], did not initiate smoking [all other response options]) |
| High number of sexual partners at 23 years | All together, in your life so far, how many people have you had sexual intercourse with? | _ _ _ people (free recall)  (Recoded into 2 categories: high [11 or more], low [<11]) |
| Trouble with the law at 24 years | Have any of these happened since you were 23 years old and did they affect you?  You were in trouble with the law. | Yes, affected me a lot  Yes, moderately affected  Yes, mildly affected  Yes, but didn’t affect me at all  No, did not happen  (Recoded into 2 categories: yes, no) |
| Ever gambled | Have you ever participated in any of the form of gambling listed* [below]? | Yes  No |
| Enjoys taking risks | Please indicate how much you agree or disagree with [this] statement.  I quite enjoy taking risks. | Agree strongly  Agree somewhat  Disagree somewhat  Disagree strongly  (Recoded into 2 categories: yes [agree], no [disagree]) |

* Tickets for the National Lottery; Scratchcards; Tickets for any other lottery; The football pools; Bingo cards or tickets; Fruit slot machines; Virtual gaming machines in a bookmaker’s to bet on a virtual roulette, keno, bingo etc.; Table games (roulette, dice or cards) in a casino; Online gambling like playing poker, bingo, slot machine style games, or casino games for money; Online betting with a bookmaker on any event or sport; Betting exchange; Betting on horse races in a bookmakers, by phone, or at the track; Betting on dog races in a bookmakers, by phone, or at the track; Betting on any other event or sport at the bookmakers, by phone or at the venue; Spread-betting; Private betting, playing cards or games for money with friends, family or colleagues; Any other form of gambling.
