# Supplementary material for: Association of genetic liability to smoking initiation with e-cigarette use in young adults: A cohort study
Source: PLoS Med. 2021 Mar 18;18(3):e1003555. doi: 10.1371/journal.pmed.1003555 (PMC7971530; doi:10.1371/journal.pmed.1003555)
Supplement: S2 Table — (DOCX) [file pmed.1003555.s004.docx]

| *p*-value thresholds | N SNPs for smoking initiation |
| --- | --- |
| 5x10^-8^ | 347 |
| 0.0005 | 2,808 |
| 0.005 | 10,098 |
| 0.05 | 42,494 |
| 0.5 | 169,364 |
