# Supplementary material for: Association of genetic liability to smoking initiation with e-cigarette use in young adults: A cohort study
Source: PLoS Med. 2021 Mar 18;18(3):e1003555. doi: 10.1371/journal.pmed.1003555 (PMC7971530; doi:10.1371/journal.pmed.1003555)
Supplement: S3 Table — (DOCX) [file pmed.1003555.s005.docx]

| Outcome  Exposure | n | OR | 95% CI | *p* |
| --- | --- | --- | --- | --- |
| Ever used e-cigarettes by 24 |  |  |  |  |
| Number of sexual partners at 23* | 2013 | 2.40 | 1.93, 2.97 | <0.001 |
| Been in trouble with the law since 23rd birthday | 2891 | 2.43 | 1.49, 3.95 | <0.001 |
| Enjoys taking risks at 24 | 2896 | 1.62 | 1.37, 1.90 | <0.001 |
| Ever gambled at 24 | 2860 | 2.05 | 1.68, 2.51 | <0.001 |
| Hyperactivity at 7 | 2340 | 1.34 | 1.12, 1.61 | 0.002 |
| Conduct disorder at 7 | 2375 | 1.24 | 1.00, 1.53 | 0.054 |
| Oppositional defiant disorder at 7 | 2380 | 1.25 | 1.04, 1.51 | 0.017 |
| Parental SEP | 2630 | 1.46 | 1.13, 1.90 | 0.004 |
|  |  |  |  |  |
| Ever initiated smoking by 24 (>100 cigarettes) |  |  |  |  |
| Number of sexual partners at 23* | 2034 | 3.21 | 2.60, 3.96 | <0.001 |
| Been in trouble with the law since 23rd birthday | 2922 | 2.12 | 1.30, 3.44 | 0.002 |
| Enjoys taking risks at 24 | 2927 | 1.78 | 1.52, 2.09 | <0.001 |
| Ever gambled at 24 | 2890 | 1.67 | 1.39, 2.02 | <0.001 |
| Hyperactivity at 7 | 2363 | 1.22 | 1.02, 1.45 | 0.032 |
| Conduct disorder at 7 | 2396 | 1.17 | 0.95, 1.45 | 0.143 |
| Oppositional defiant disorder at 7 | 2407 | 1.23 | 1.02, 1.47 | 0.026 |
| Parental SEP | 2655 | 1.59 | 1.24, 2.05 | <0.001 |

*Low (<11) vs. high (11 or more) number of sexual partners.
