# Supplementary material for: Association of genetic liability to smoking initiation with e-cigarette use in young adults: A cohort study
Source: PLoS Med. 2021 Mar 18;18(3):e1003555. doi: 10.1371/journal.pmed.1003555 (PMC7971530; doi:10.1371/journal.pmed.1003555)
Supplement: S4 Table — (DOCX) [file pmed.1003555.s006.docx]

| Outcome  *p*-value threshold | n | beta | 95% CI | *p* |
| --- | --- | --- | --- | --- |
| Ever e-cigarette use by 24 (restricted to never smokers*) | 1037 |  |  |  |
| 5x10^-8^ |  | 1.28 | 0.94, 1.74 | 0.111 |
| 0.0005 |  | 1.10 | 0.81, 1.48 | 0.550 |
| 0.005 |  | 1.27 | 0.94, 1.71 | 0.124 |
| 0.05 |  | 1.39 | 1.02, 1.90 | 0.039 |
| 0.5 |  | 1.57 | 1.16, 2.12 | 0.004 |

*Never smokers in this analysis were defined as never having smoked a whole cigarette in their lifetime.
