# Supplementary material for: Association of genetic liability to smoking initiation with e-cigarette use in young adults: A cohort study
Source: PLoS Med. 2021 Mar 18;18(3):e1003555. doi: 10.1371/journal.pmed.1003555 (PMC7971530; doi:10.1371/journal.pmed.1003555)
Supplement: Attachment — Submitted filename: PMEDICINE-D-20-02674_R1_reviewer.docx [file pmed.1003555.s007.docx]

**Title**: Association of Genetic Liability to Smoking Initiation with E-Cigarette Use in Young Adults

**Manuscript Number**: PMEDICINE-D-20-02674R1

**Reviewer Competing Interest Statement:** I report no competing interests.

**Anonymity:** I would like to remain anonymous.

**Reviewer Comments:**

This is an interesting report that outlines how the authors determined the extent to which smoking initiation and e-cigarette use have a shared genetic etiology. Specifically, the authors aimed to investigate whether polygenic risk scores for smoking initiation are associated with ever use of e-cigarettes in young adulthood. The authors also explore associations with outcomes that are not plausibly biologically related or that precede smoking. Results suggest that smoking initiation PRS is associated with both e-cigarette use and general risk taking behavior (as determined by analyses involving negative controls). This is definitely a research topic that is of growing interest in the field, and the authors do a good job of explaining concepts throughout the text. However, there are a few things that require additional clarification::

**Major points:**

- Statistical analyses were adjusted for age, sex, and the first 10 principal components. Did the authors also conduct sensitivity analyses without adjustment for age, since cigarette and e-cigarette use outcomes are reported at a specific age (e.g. age 23-24)? Or, can the authors provide an explanation for why they needed to additionally adjust models for age, given that the outcomes are based on behavior at a specific age?
- It is noted on page 15 that there was evidence of a positive association between smoking initiation PRS and hyperactivity at 7 years, but not at the most stringent threshold - why might this be?
- In addition to stating that results within this study differ than that of Allegirini and colleagues and moving on to describe what might explain associations between smoking initiation PRS and e-cigarette use, it would be useful to also include information on why the authors think that their results differ from that found by Allegirini and colleagues (e.g. is it due to population characteristics, study design differences, etc.)? This has implications for future research examining shared genetic etiologies between smoking and e-cigarette use.
- Have any follow-up analyses been conducted to investigate how controls (e.g. positive and negative) influence associations between polygenic risk scores for smoking initiation and e-cigarette use? This seems to be the next logical step, since there is mention in the Introduction section that “some of the genetic influence on smoking initiation is mediated by... risk-taking”. I think including these analyses would help to strengthen the paper, as it will provide a clearer picture of associations between smoking PRS, risk taking behavior, and e-cigarette use.

**Minor points:**

- There are a few places where the citations are not in a singular citation style (e.g. in the introduction, there are number citations [2,3] as well as APA style citations (e.g. Khouja, Taylor, and Muafo, 2019). Please check through the manuscript to make sure that the journal style of referencing is consistent.
- It might be helpful to readers to include a figure that visually displays the different models for shared genetic liability (e.g. vertical pleiotropy, horizontal pleiotropy, etc.), as well as which model was tested in this specific study.
- On page 5, the following sentence needs to be revised for clarity: “Alternatively, triangulation could aid consideration of whether an association is due to a shared genetic predisposition between parents and offspring; where parents share their offspring’s smoking initiation predisposition and consequently expose their offspring to cigarette smoke in utero or in childhood, an apparent effect of a child’s own genetic 94 variants may be a result of their pre-natal or post-natal environment due to their parents’ genetic variants.” I would suggest shortening the sentence into smaller sentences.
- Is there a particular reason why the authors chose to focus on the age group 23-24 years? If so, this information would be useful to include.
- On page 9, please indicate that the acronym parental SEP is socioeconomic position.
- On page 10, the threshold for a high number of sexual partners at 23 is noted to be 11 or more sexual partners - how did the authors derive this threshold?
