# Supplementary material for: Association of genetic liability to smoking initiation with e-cigarette use in young adults: A cohort study
Source: PLoS Med. 2021 Mar 18;18(3):e1003555. doi: 10.1371/journal.pmed.1003555 (PMC7971530; doi:10.1371/journal.pmed.1003555)
Supplement: Attachment — Submitted filename: PMEDICINE-D-20-02674R2 Response 210117.docx [file pmed.1003555.s009.docx]

We are very grateful for the comments from the reviewers and editors. As described below, we have addressed the remaining editorial and production issues.

1. Please ensure that the study is reported according to the STROBE guideline, and include the completed STROBE checklist as Supporting Information. Please add the following statement, or similar, to the Methods: "This study is reported as per the Strengthening the Reporting of Observational Studies in Epidemiology (STROBE) guideline (S1 Checklist)."

The STROBE guideline can be found here: <http://www.equator-network.org/reporting-guidelines/strobe/>

When completing the checklist, please use section and paragraph numbers, rather than page numbers.

RESPONSE: We have now included a STROBE checklist in the supplementary material (S1 Checklist). We have also referred to this checklist in the manuscript at the beginning of the methods section (line 160).

2. You note that your analyses were pre-planned but I didn’t see an analysis plan in the SI files. If one exists (e.g., in an IRB or grant application), please include this in the SI files and reference it from the Methods section. If no such document exists, please note this in the Methods section.

RESPONSE: We have clarified that the analysis plan was not published prior to data analysis:

“These analyses were planned prior to the analysis being conducted and were not data driven; however, the plan was not made publicly available prior to the analysis” (line 246).

3. Perhaps "Tobacco smoking ..." at line 17, and “tobacco smokers” at line 72?

RESPONSE: We have amended these sentences as suggested (line 17 and 71).

4. Line 45, remove “Taken together”.

RESPONSE: We have removed “taken together” from this sentence.

5. It seems that you use “e-cigarette use” and “vaping” interchangeably; perhaps state "e-cigarette use or 'vaping'" early on? At line 65, do you mean "... both vaping and using cigarettes ..."?

RESPONSE: Given that the definition of vaping varies, we have removed the words ‘vaping’ and ‘vaped’ and used consistent terminology throughout (e.g., e-cigarette use).

6. Please begin the Discussion with a brief description of what was done before summarizing results.

RESPONSE: We have now included the following sentences at the beginning of the discussion:

“In this study, we explored the association between smoking initiation PRS and e-cigarette use, using logistic regression. We further explored the findings by observing the association between smoking initiation PRS and positive controls (smoking) and negative controls (e.g., risk taking), as well as restricting the analysis to never smokers” (line 336).

7. In table 3, perhaps add more contest in the “Number of sexual partners by 23” outcome, to avoid misinterpretation of “n=2,505" one column over.

RESPONSE: We have amended this outcome to read:

“11 or more sexual partners by age 23” (line 302).

8. Please remove the funding and COI statement can go from the main text and make sure this information is captured in the appropriate sections of the submission form.

RESPONSE: We have removed the funding and COI statement and ensured this information is accurately reflected in the submission form.

9. References 1 and 19 look incomplete; Ref 3 needs reformatting and the journal name; Ref 4, 28, should note that they’re preprints.

RESPONSE: Thank you for highlighting these Endnote formatting errors. References 1 and 19 have been updated and reference 3 has been reformatted. References 4 and 20 have now been published, so the published version is now referenced. References 27 and 28 have been updated to clarify that they are preprints.
