# Supplementary material for: Association of genetic liability to smoking initiation with e-cigarette use in young adults: A cohort study
Source: PLoS Med. 2021 Mar 18;18(3):e1003555. doi: 10.1371/journal.pmed.1003555 (PMC7971530; doi:10.1371/journal.pmed.1003555)
Supplement: Attachment — Submitted filename: PLOSMED Reviewer Comments and Response_201029.docx [file pmed.1003555.s008.docx]

We thank the editor and reviewers for their comments and have addressed these as detailed below.

Requests from the editors:

1. Thank you for noting the restrictions on data sharing. Please edit the statement to read:

“The data used in this study are available on request to the ALSPAC Executive (alspac-exec@bristol.ac.uk). The ALSPAC data management plan describes in detail the policy regarding data sharing. Full instructions for applying for data access can be found

here: http://www.bristol.ac.uk/alspac/researchers/access/. The ALSPAC study website

contains details of all the data that are available (http://www.bristol.ac.uk/alspac/researchers/our-data/).”

RESPONSE: We have amended the statement as requested.

2. Please revise your title according to PLOS Medicine's style. Please place the study design (e.g, "a cohort study”) in the subtitle (ie, after a colon).

RESPONSE: We have revised our title to: “Association of genetic liability to smoking initiation with e-cigarette use in young adults: A cohort study”.

3. In the Abstract Methods and Findings, Please include the study design, population and setting, number of participants and relevant demographics (e.g., mean age, sex, race/ethnicity), years during which the study took place, length of follow up, and main outcome measures.

RESPONSE: We have amended the abstract methods and findings to ensure these details are stated (page 2, line 22).

4. In the Abstract, and throughout the manuscript (Results and Tables), please include p values alongside 95% CIs for comparisons.

RESPONSE: We now include p-values in the abstract and throughout the manuscript, alongside confidence intervals.

5. In the Abstract Methods and Findings, please include the actual amounts and/or absolute risk(s) of relevant outcomes, not just relative risks or correlation coefficients. (example for absolute risks: PMID: 28399126).

RESPONSE: We have added the following sentence to the abstract:

“A total of 878 young adults (30%) had ever vaped at 24 years, and 150 (5%) were regular vapers at 24 years” (page 2, line 30).

6. Abstract, line 31: please define the p-value thresholds used for these associations and include a bit more explanation.

RESPONSE: We have clarified that the results reflect the association with the smoking polygenic risk score created using the genome-wide significance threshold, and have added the following explanation:

“We observed positive associations of similar magnitude between smoking initiation PRS (created using the p < 5×10^-8^ threshold) and both smoking initiation (OR = 1.29, 95% CI 1.19 to 1.39, p < 0.001) and ever e-cigarette use (OR = 1.24, 95% CI 1.14 to 1.34, p < 0.001) by the age of 24 years, indicating that a genetic predisposition to smoking initiation is associated with an increased risk of vaping” (page 2, line 31).

7. In the last sentence of the Abstract Methods and Findings section, please describe the main limitation(s) of the study's methodology.

RESPONSE: At the end of the abstract methodology we have included:

“However, this study is limited by the relatively small sample size and potential for collider bias” (page 3, line 40).

8. At this stage, we ask that you include a short, non-technical Author Summary of your research to make findings accessible to a wide audience that includes both scientists and non-scientists. The Author Summary should immediately follow the Abstract in your revised manuscript. This text is subject to editorial change and should be distinct from the scientific abstract. Please see our author guidelines for more information: https://journals.plos.org/plosmedicine/s/revising-your-manuscript#loc-author-summary

RESPONSE: We have added a short author summary following the abstract (page 3, line 47):

Why Was This Study Done?

- Some individuals are more likely to smoke due to their genetics, but little is currently known about the genetic influences on e-cigarette use.
- Given that many people who use e-cigarettes have smoked before, it is likely that there may be an overlap between genetic influences on smoking and e‑cigarette use.
- Such an overlap may explain why people who use e-cigarettes but have not smoked before are more likely to go on to start smoking later.

What Did the Researchers Do and Find?

- We examined the association between genetic variants associated with smoking initiation and both e-cigarette use and risk-taking behaviour in a cohort of 2,894 young adults.
- Our results indicate that the genetic factors that influence smoking initiation are similarly related to e-cigarette use and risk-taking behaviours.

What Do These Findings Mean?

- Smoking may cause people to use e-cigarettes (i.e., to stop smoking), but there may also be an underlying genetic predisposition to risk-taking which influences the likelihood that someone will both smoke and use e-cigarettes
- The findings could have important implications for policy – if young people are predisposed to both smoking and using e-cigarettes, bans which aim to prevent e cigarette use may encourage smoking where only cigarettes are available.”

9. Did your study have a prospective protocol or analysis plan? Please state this (either way) early in the Methods section.

a) If a prospective analysis plan (from your funding proposal, IRB or other ethics committee submission, study protocol, or other planning document written before analyzing the data) was used in designing the study, please include the relevant prospectively written document with your revised manuscript as a Supporting Information file to be published alongside your study, and cite it in the Methods section. A legend for this file should be included at the end of your manuscript.

b) If no such document exists, please make sure that the Methods section transparently describes when analyses were planned, and when/why any data-driven changes to analyses took place.

c) In either case, changes in the analysis-- including those made in response to peer review comments-- should be identified as such in the Methods section of the paper, with rationale.

RESPONSE: We did not pre-register our study protocol or analysis plan; we have clarified which analyses were pre‑planned in the methods section of the manuscript (page 12, line 245).

10. Please call out the ethics section in the Methods with its own subheading. Additionally, please specify if consent was written or oral.

RESPONSE: We have included a subheading for the ethics section (page 9, line 193) and amended the paragraph to include that written informed consent was provided.

11. Methods, Line 148: at what time?

RESPONSE: We have clarified that consent was taken “following the recommendations of the ALSPAC Ethics and Law Committee at the time of study initiation (i.e., 1991)”.

12. Please present and organize the Discussion as follows: a short, clear summary of the article's findings; what the study adds to existing research and where and why the results may differ from previous research; strengths and limitations of the study; implications and next steps for research, clinical practice, and/or public policy; one-paragraph conclusion.

RESPONSE: We have reorganized the discussion in line with these recommendations.

Reviewer #1:

1. The primary factor missing from the results is an overall understanding of the types of genetic factors that may contribute to the findings. Thus, a more comprehensive description of the makeup of the genes that contribute to the polygenic risk scores would be helpful.

RESPONSE: We have included a brief comment on the genes contributing to the score (page 9, line 176). Most of the variants were intergenic with no known function; a comprehensive description of the genetic variants contributing to this score, and the genes they are within (e.g., *PPP1R1B, GRIN2A, HOMER2*), can be found in Liu et al. (2019).

2. Also, of the 378 single nucleotide polymorphisms that went into the smoking initiation polygenic risk scores, could the authors identify which of these genetic variants have previously been identified as functional? A brief discussion of the relevance of the outcome would be helpful for the reader/reviewer.

RESPONSE: As we note above, we have directed the readers to the original GWAS paper (Liu et al., 2019) where there is a more comprehensive discussion of the genes influencing smoking initiation, including functional genes. Very few of the SNPs included in the polygenic risk score have a known function, which is why we are particularly interested in the specificity of the score, but glutamate and dopaminergic gene pathways were enriched for smoking initiation. Also, the rs6265 variant (a non-synonymous SNP in the BDNF gene) which has previously been found to be associated with smoking initiation [14] was also associated with smoking initiation in GSCAN (page 9, line 172).

3. Further, could the authors comment on how important their findings are when OR are between OR = 1.24 - 1.29? How is this similar or different if only one genetic polymorphism was evaluated at the most significant *p*-value vs. smoking initiation PRS, for example?

RESPONSE: Given the small amount of variation in smoking initiation explained by the SNPs (2.3%), and the fact that any causal effect will only explain a proportion of the outcome, small effect sizes are to be expected (page 18, line 332). There is no one single SNP that predicts a large proportion of variance in smoking initiation, therefore, our score should better predict smoking initiation than any single SNP.

Reviewer #2:

1. The authors showed the some associations between PRS and e-smoking, and also some associations between PRS and smoking. However, the 3-way relationship has not been systematically untangled. To account for the smoking impact between the PRS and e-smoking, for example, mediation analysis could be used while smoking is the mediator.

RESPONSE: Although a mediation analysis could help to systematically untangle the potential 3-way relationship between the PRS, smoking initiation and e-cigarette use, we do not think this analysis would be appropriate given the nature of our sample.

First, there would be limited power to conduct a mediation analysis as there are too few never smoking e-cigarette users in this sample (n = 46 had never smoked 1 cigarette or more and n = 257 never smoked 100 cigarettes or more in their lifetime).

Second, the association found between smoking initiation PRS and externalising disorders at age 7 years (before the majority of young people had initiated smoking) also supports a common liability interpretation, suggesting the relationship is not solely mediated via smoking initiation.

Third, a mediation analysis would assume that there is no unmeasured confounding and, given our results suggest that the smoking initiation PRS may predict risk-taking in general, we do not think it would be appropriate to assume this.

Therefore, we do not think a mediation analysis would be appropriate. Ideally, future research would allow us to identify separate SNPs for smoking initiation and e-cigarette use which would then allow us to conduct a multivariable Mendelian randomisation analysis that could help us investigate this relationship further. We have added a brief discussion of this to page 22 (line 434).

2. Not convinced why different P-value thresholds were used. The 5x10^-8^ one ended up with 347 SNPs should be used as it took account of proper multiple test adjustment. All the others are not adequate and irrelevant.

RESPONSE: We have used varying thresholds in order to explore whether pleiotropy may drive any associations observed. As discussed in the manuscript, the more SNPs included in a PRS, the less likely it is that every SNP will be truly associated with the trait of interest, and the more likely it is that some SNPs will be associated with other (horizontally pleiotropic) traits. Therefore, when the most stringent *p*-value threshold is used to determine inclusion of SNPs in the score, the polygenic risk score is more specific to smoking initiation and includes fewer horizontally pleiotropic SNPs. However, when we use less stringent thresholds the score may explain more variance in smoking initiation, but we also increase the likelihood of including SNPs which relate to other traits (i.e., are horizontally pleiotropic). Thus, observing increasing magnitudes of effect as the threshold is relaxed in the negative control analyses indicates that more pleiotropy is being reintroduced. When using these varying polygenic risk score thresholds to explore the association with negative control outcomes (e.g., externalising disorders in childhood) we aim to see how specific the PRS must be before it no longer predicts these pleiotropic traits. If we observe similar magnitudes of effect when exploring the association between the negative control outcomes and the polygenic risk scores at varying thresholds, it would indicate that the more specific polygenic risk score is driving the effect and that pleiotropy is not influencing the scores. Therefore, using varying thresholds can improve our understanding of whether pleiotropy may drive any associations observed. We have clarified this in the text (page 7, line 144)

3. It said in a few places that the analyses were adjusted by principal components 1-10. But principal components of what? Never explained anywhere in the paper.

RESPONSE: We have clarified in the text that these are the first 10 “principal components of population stratification” (page 11, line 238).

4. Still not clear and convinced what all these negative controls were for.

RESPONSE: Given the possibility of a shared liability mechanism (e.g., an underlying risk-taking phenotype), we wanted to explore a range of risk behaviours. We explored factors at a similar age range to e-cigarette use and smoking with varied intensity of risk-taking, but these could arguably be a causal effect of smoking. Therefore, we also explored impulsivity in childhood, at an age prior to the initiation of smoking (which therefore cannot reflect a causal effect of smoking). Finally, we explored whether the smoking initiation PRS may be capturing other social influences on smoking (e.g., socioeconomic position at birth) which cannot plausibly have been a causal effect of the young adult’s own smoking (although there could be a dynastic effect of parental smoking, which is correlated with offspring genotype). We have clarified this in the text (page 8, line 151).

5. Description of the datasets being used and selected was difficult to follow and confusing.

RESPONSE: We have rephrased these paragraphs for greater clarity (page 8, line 161).

6. Statistics analyses section is too brief and could be more comprehensive and detailed. By the way, what are the 'first 10 principal components'?

RESPONSE: As stated above, we have clarified the that the principal components are of population stratification. We have also added further details to the statistical analyses section (page 11, line 236).

Reviewer #3:

1. Limitations also include variability in the nature of key assessments and the use of self-report data, which should be mentioned.

RESPONSE: We have added these limitations to the discussion (page 21, line 407).

2. More information about the potential policy implications of the results should be offered.

RESPONSE: We have added a paragraph discussing the potential policy implications of these results on page 22, line 439. The implications relate to the impact of strict regulations (e.g., bans) which may unintentionally increase smoking rates.

Reviewer #4:

1. Statistical analyses were adjusted for age, sex, and the first 10 principal components. Did the authors also conduct sensitivity analyses without adjustment for age, since cigarette and e-cigarette use outcomes are reported at a specific age (e.g. age 23-24)? Or, can the authors provide an explanation for why they needed to additionally adjust models for age, given that the outcomes are based on behavior at a specific age?

RESPONSE: Although the majority of participants completed the questionnaires at the given age stated (e.g., 24 years), there was some variation in the age of completion. At the age 24 questionnaire, for example, there was a difference of 32 months between the youngest and oldest participants. Therefore, we included adjustment for age in months.

2. It is noted on page 15 that there was evidence of a positive association between smoking initiation PRS and hyperactivity at 7 years, but not at the most stringent threshold - why might this be?

RESPONSE: As discussed in our introduction, the more SNPs included in a PRS, the less likely it is that the effect of each variant on the trait of interest is proportional to the effect of the trait of interest on the exposure, and the more likely it is proportional to the effects on other traits (i.e., horizontal pleiotropy). Therefore, it is likely that we do not see an association between smoking initiation polygenic risk scores and hyperactivity when the most stringent *p*-value threshold is used to determine inclusion of SNPs in the score because this polygenic risk score is more specific to smoking initiation and includes fewer horizontally pleiotropic SNPs. As we use less stringent thresholds, we increase the likelihood of including SNPs which relate to a broader risk-taking phenotype, thus we observe stronger evidence of an association between these polygenic risk scores an impulsivity in childhood. As we have stated in the discussion, increasing magnitudes and strengthened evidence of association with PRS and negative controls at less stringent *p*-value thresholds suggests that the smoking initiation polygenic risk score is capturing, at least in part, a broad phenotype which is not entirely specific to smoking/nicotine. This argument is strengthed where we observe some evidence of an association between negative controls and the smoking initiation polygenic risk score even at the most stringent *p*-value threshold for inclusion in the score.

3. In addition to stating that results within this study differ than that of Allegirini and colleagues and moving on to describe what might explain associations between smoking initiation PRS and e-cigarette use, it would be useful to also include information on why the authors think that their results differ from that found by Allegirini and colleagues (e.g. is it due to population characteristics, study design differences, etc.)? This has implications for future research examining shared genetic etiologies between smoking and e-cigarette use.

RESPONSE: The differences could be due to the differences in polygenic scores used – our score was based on the results of a much larger genome-wide association study, with more statistical power to detect associated genetic variants. We have amended the discussion to consider why the differences between our results and the results of Allegrini and colleagues (page 18, line 337).

4. Have any follow-up analyses been conducted to investigate how controls (e.g. positive and negative) influence associations between polygenic risk scores for smoking initiation and e-cigarette use? This seems to be the next logical step, since there is mention in the Introduction section that “some of the genetic influence on smoking initiation is mediated by... risk-taking”. I think including these analyses would help to strengthen the paper, as it will provide a clearer picture of associations between smoking PRS, risk taking behaviour, and e-cigarette use.

RESPONSE: We appreciate that this is the logical next step, but given the reasons outlined above (in response to reviewer 2, question 1), we do not think it is appropriate to conduct a mediation analysis in this sample.

5. There are a few places where the citations are not in a singular citation style (e.g. in the introduction, there are number citations [2,3] as well as APA style citations (e.g. Khouja, Taylor, and Munafo, 2019). Please check through the manuscript to make sure that the journal style of referencing is consistent.

RESPONSE: Thank you for pointing out this error, we have amended the style where incorrect.

6. It might be helpful to readers to include a figure that visually displays the different models for shared genetic liability (e.g. vertical pleiotropy, horizontal pleiotropy, etc.), as well as which model was tested in this specific study.

RESPONSE: We have now included a figure (Figure 1) which displays the models as suggested.

7. On page 5, the following sentence needs to be revised for clarity: “Alternatively, triangulation could aid consideration of whether an association is due to a shared genetic predisposition between parents and offspring; where parents share their offspring’s smoking initiation predisposition and consequently expose their offspring to cigarette smoke in utero or in childhood, an apparent effect of a child’s own genetic 94 variants may be a result of their pre-natal or post-natal environment due to their parents’ genetic variants.” I would suggest shortening the sentence into smaller sentences.

RESPONSE: We have revised the sentence to:

“Alternatively, triangulation could help us consider whether an association is due to a shared genetic predisposition between parents and offspring. Where parents share their offspring’s smoking initiation predisposition, they are likely to expose their offspring to cigarette smoke in utero or in childhood. Consequently, an apparent effect of a child’s own genetic variants may be a result of their pre-natal or post-natal environment due to a dynastic effect of their parents’ genetic variants” (page 6, line 119).

8. Is there a particular reason why the authors chose to focus on the age group 23-24 years? If so, this information would be useful to include.

RESPONSE: We chose to use the most detailed and recent available data in the Avon Longitudinal Study of Parents and Children, a prospective cohort with extensive data from birth to young adulthood with available genetic data. We have clarified this in the text (page 9, line 178).

9. On page 9, please indicate that the acronym parental SEP is socioeconomic position.

RESPONSE: The acronym of socioeconomic position (SEP) is indicated earlier in the manuscript (page 6, line 113).

10. On page 10, the threshold for a high number of sexual partners at 23 is noted to be 11 or more sexual partners - how did the authors derive this threshold?

RESPONSE: As stated in the Supplementary Material, “a high number of sexual partners at 23 years was determined using the upper quartile for number of lifetime sexual partners in the ALSPAC sample (11 or more sexual partners).” We direct readers to the supplementary material at the end of the paragraph discussing the negative controls in the methods section. We have clarified this in the footnotes of Table 1 and Table 3.
